# Effect of Process Parameters on the Graphite Expansion Produced by a Green Modification of the Hummers Method

**DOI:** 10.3390/molecules27217399

**Published:** 2022-10-31

**Authors:** Gabriela Tarango-Rivero, José M. Mendoza-Duarte, Audel Santos-Beltrán, Ivanovich Estrada-Guel, Carlos G. Garay-Reyes, Pedro Pizá-Ruiz, Cynthia D. Gómez-Esparza, Enrique Rocha-Rangel, Roberto Martínez-Sánchez

**Affiliations:** 1 Centro de Investigación en Materiales Avanzados, CIMAV, Miguel de Cervantes 120, Chihuahua 31136, Mexico; 2 Centro de Tecnología Avanzada (CIATEQ), Eje 126 225, Industrial San Luis, San Luis 78395, Mexico; 3 Departamento de Nanotecnología, Universidad Tecnológica de Chihuahua Sur, Km. 3.5 Carr. Chihuahua-Aldama, Chihuahua 31313, Mexico; 4 Facultad de Ingeniería, Universidad Autónoma de Chihuahua, Chihuahua 31125, Mexico; 5 Subsecretaría de Inteligencia y Análisis Policial, Secretaría de Seguridad Pública del Estado, Chihuahua 31313, Mexico; 6 Departamento de Investigación y Posgrado, Universidad Politécnica de Victoria, Ciudad Victoria 87138, Mexico

**Keywords:** milling, graphite, exfoliation, green chemistry, Hummers

## Abstract

Adsorption stand out among other standard techniques used for water treatment because of its remarkable simplicity, easy operation, and high removal capability. Expanded graphite has been selected as a promising agent for oil spill adsorption, but its production involves the generation of corrosive remnants and massive amounts of contaminated washing waters. Although the advantageous use of the H_2_O_2_–H_2_SO_4_ mixture was described in 1978, reported works using this method are scarce. This work deals with the urgent necessity for the development of alternative chemical routes decreasing their environmental impact (based on green chemistry concepts), presenting a process for expanded graphite production using only two intercalation chemicals, reducing the consumption of sulfuric acid to only 10% and avoiding the use of strong oxidant salts (both environmentally detrimental). Three process parameters were evaluated: milling effect, peroxide concentration, and microwave expansion. Some remarkable results were obtained following this route: high specific volumes elevated oil adsorption rate exhibiting a high oil–water selectivity and rapid adsorption. Furthermore, the recycling capability was checked using up to six adsorption cycles. Results showed that milling time reduces the specimen’s expansion rate and oil adsorption capacity due to poor intercalant insertion and generation of small particle sizes.

## 1. Introduction

An oil spill is an accidental or deliberate release of solid or liquid hydrocarbons into the environment [1]. Although hydrocarbons are present in various types and forms, they are generally problematic to remove, causing catastrophic effects on ecosystems if effective treatment is not correctly applied. Thus, the treatment of oil spills must be efficient and timely to avoid further pollution aggravation [2]. Diesel fuel is a major energy source worldwide and one of the most widespread contaminants in soil and water [3]. For oily water treatment, membrane systems and advanced oxidation are used; however, there are limitations due to high capital and maintenance costs. Chemical flocculation has high removal efficiency, but it consumes huge amounts of energy. Biological treatment is also used despite requiring a further consuming time treatment. A practical alternative for water treatment is the adsorption process; the main mechanism is accumulating a layer of solute molecules on the solid surface in contact [4]. Adsorption is envisaged as a promising technique due to its simplicity, ease of operation, high removal capability [5], and accelerated removal of pollutants [6]. Some materials have been proposed for adsorption agents, such as nanomaterials, nanocomposites, nanoparticles, clays, biopolymers, metal-organic frameworks, and zeolites [4]. Still, porous carbon materials are widely used in wastewater treatment due to their micropores and excellent adsorption capacity [7]. From carbon-based materials, activated carbon has been recommended as an ideal agent for contaminants removal in water. Still, its production and regeneration are quite expensive [8]. The same happened with special molecules such as carbon nanotubes and graphene, which have successfully proven their extraordinary performance but, at present, are too expensive to be used in oil clean-up [9]. Expanded graphite (EG) has been receiving global attention due to the number of potential applications based on its intrinsic properties, such as low density, high porosity, and electrical conductivity, making it a promising material for fuel cells, electromagnetic interference shielding, catalyst, vibration damping, supercapacitors, biomedical materials and, as spilled oil adsorbent [10,11,12]. The high effectiveness of EG for oil on water or pure oil sorption was early described by Inagaki and Toyoda two decades ago [13]. The classical method for the EG preparation using chemical reactions is cheap and easily scalable, but it requires the mandatory use of highly concentrated mineral acids with strong oxidizing agents for the graphite intercalation process [11]; these chemicals and their corresponding reaction remnants can cause adverse environmental effects if they are not disposed of properly. An ideal adsorbent must not only show high efficiency and mechanical strength for practical use, but it also needs an adequate balance with some features such as low cost and high biodegradability; because it is important to preserve the adsorption performance under requirements such as sustainability and green remediation, where the main idea is to replace the use of toxic reagents with lower impact ones [1]. This is in good agreement with a concept developed in the business and regulatory communities as a natural evolution of pollution prevention initiatives, called green chemistry. It deals with the design of chemical products and processes that reduce or eliminate the use or generation of hazardous substances and applies across the life cycle of a chemical product, including its design, manufacture, use, and ultimate disposal [14]. Following these principles, an alternative chemical route is described; this route uses a single mixture of H_2_O_2_–H_2_SO_4_. Although the advantageous use of this method was described in 1978 [15], reported works using this route are still scarce [7,16,17]. Even though the above patent recommends using a sulfuric-graphite paste using fine graphite particles for an efficient intercalation process, the effect of comminution was not adequately described. In this aspect, high energy ball milling (HEBM) is identified as a sustainable dry technique (solvent-free) that can be advantageously used to increase the reaction yield. In HEBM, the highly energized balls transfer energy to the powders, modifying the graphitic structure; in the presence of chemicals, some functional groups on the edges, surfaces, and basal planes of graphite can be introduced. HEBM not only causes particle comminution but also refines the grains into nanoscale sizes, increasing the proportion of highly active regions [18]. This work deals with a green chemistry-based method for EG production, using a single mixture of intercalation chemicals (easily available and inexpensive); low sulfuric acid and hydrogen peroxide consumption is also prioritized. The used H_2_SO_4_–H_2_O_2_ couple eliminates the common use of transition metals salts as permanganate or dichromate (like Hummers and Chandra chemical routes), generating a cleaner and simpler process. The effect of HEBM to activate and generate finer graphite particles, the H_2_O_2_ concentration, and microwave expansion were also evaluated. Finally, the prepared EGs were tested for diesel absorption in an oil–water mixture, showing high oil selectivity and adsorption rates above the reported in the literature.

## 2. Results

### 2.1. Powders Characterization

**Figure 1:**
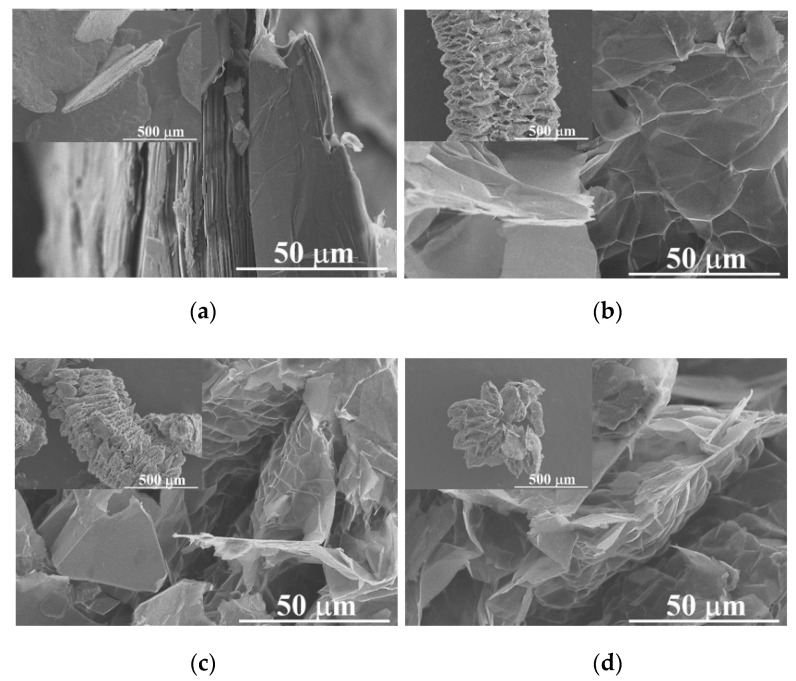
SEM micrographs (100 and 1000X) of a single particle of (**a**) raw graphite (NGr) and intercalated products (**b**) 0–2, (**c**) 5–2, and (**d**) 15–2.

**Figure 2:**
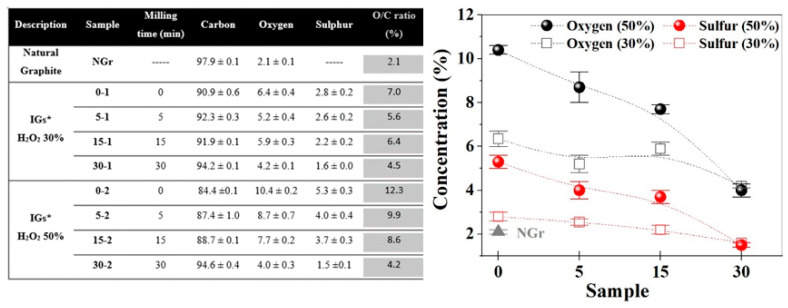
Summary chart with samples nomenclature, elemental results (in weight %), carbon/oxygen ratio, and their respective EDS plot. * The intercalated graphites (IGs) were prepared using two hydrogen peroxide solutions of different concentrations.

Scanning electron microscopy (SEM). The morphological features of the original graphite and changes in the intercalated samples (using 30 y 50% H_2_O_2_) were studied through this technique; Figure 1a shows the intrinsic lamellar structure of the raw graphite powders (NGr), a close magnification performed on a lateral border resolves the good packet graphitic sheets [19]. After the intercalation process, this packet array is transformed into a vermicular structure due to a preferential expansion along the c-axis of the graphite structure [10]. In Figure 1b–d, we notice several grooves generated at the expense of the original graphitic layers. This condition distorts the graphene layers due to the linkage of multiple oxygen species [20]. It was reported that this condition provides channels to facilitate the transport of fluids during the reactions [11]. Chemical oxidation reactions can weaken the attraction forces by increasing the spacing between adjacent layers, and functional groups rich in oxygen, such as hydroxyl and epoxy, can be inserted into graphite layers [21] or even in the external graphite surface [22]; both reactions induced by hydrogen peroxide in the presence of sulfuric acid. It was mentioned that the morphology and pore structure of EG can be determined by altering preparation conditions [11]. Graphite intercalation (IG) results from the insertion of different chemical species called intercalants between host graphite sheets which can be expanded by heating [21]. In our case, we can notice a remarkable expansion of intercalated samples even before the heating process; this can be related to the instability of H_2_O_2_, resulting in gas formation between graphitic layers [23,24]. Milling induces strong comminution of graphite particles; thus, the length and size of isolated worms are smaller with further milling, as can be seen in Figure 1b–d. Some compositional analyses (SEM-EDS) were performed (in triplicate) on the intercalated samples at low magnifications (25X) on a thick compressed pellet (to reduce the holder interaction signal). It can be noticed that EDS results of IGs show no metallic impurities because this processing method avoids the use of metallic oxidants in the form of sodium nitrate, potassium permanganate, or dichromate; the above simplifies the washing procedure by using lesser washing steps and reducing the volume of washing waters. IGs are composed mainly of carbon, oxygen, and sulfur (C, O, and S). In the case of O, there is a notable increase in its content; this indicates that polar oxygen groups were introduced during the intercalation process, as was mentioned by Zhao et al. [11]. Figure 2 shows the chemical composition of the graphites before and after the intercalation process; the EDS plot exhibits a clear effect of composition changes as a function of H_2_O_2_ concentration and milling time. Flake graphite is a naturally occurring mineral purified to remove heteroatomic contamination [25]. Thus, O presence may be related to purification process remnants. As can be seen, the O/C ratio increased from 2.1 for the raw graphite to 12.3 for the 0–2 sample. However, the 0–1 sample shows a lower value of 7.0; this can be related to the effect of hydrogen peroxide concentration; higher H_2_O_2_ concentration means more oxygen available and consequently higher graphite oxidation [26,27]. Additionally, is noticeable a clear reduction of O and S contents in samples with further milling; a possible explanation is related to the graphite particle size: the intercalated molecules are preferentially bonded between the interlayer gallery by diffusion mechanisms, but these chemical groups are weakly bonded to the basal planes of graphite surface. In our case, the high-energy milling of brittle materials can induce a further particle reduction, increasing the ratio of surface/mass and reducing the internal zones where the intercalation molecules can be attached. Thus, the available space for intercalation is reduced, so the concentration of O and S.

**Figure 3:**
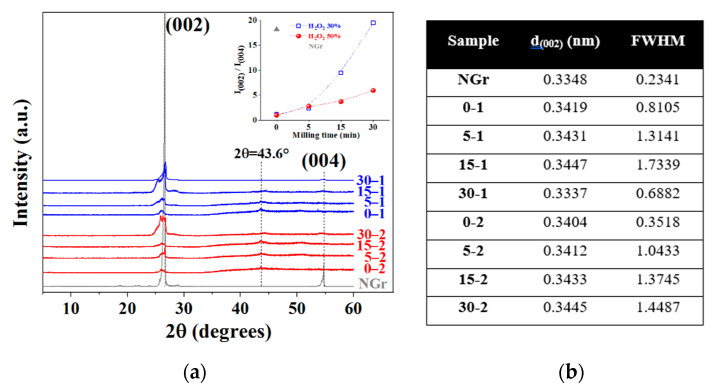
XRD patterns of samples with relative intensities (002)/(004) plot (**a**), calculated d spacing and full width at half maximum observed values (**b**).

X-ray diffraction (XRD). Some studies were performed to determine the crystallinity and follow the interlayer spacing modification of graphite and the intercalated graphites. Figure 3a shows the diffractograms of the IGs compared to NGr; we can notice a notable effect of the milling time and H_2_O_2_ concentration on the sample structure. NGr sample has a sharp and intense peak located at 2θ = 26.60° (d = 0.3348 nm), which represents the reflections in the perpendicular direction (c-axis) of the graphite hexagonal planes and is commonly correlated to the normal graphite spacing between the (002) graphite planes, the other important peak is observed at 2θ = 54.76°, this corresponds to the (004) plane. On the contrary, IGs diffractograms show notable changes in their profiles, such as broadening and intensity loss in the (002) peak due to crystallinity loss after the intercalation process. Peak broadening is related to lattice distortion in the AB stacking order of the graphite lattice because oxidation can reduce the periodic graphitic crystalline arrangement. Although the exact structure is difficult to determine, it is clear that the original contiguous graphitic lattice is interrupted by the presence of radicals and oxygenated organic groups; this is reflected in an increase in interlayer spacing from 0.335 (graphite) to more than 0.625 nm [25]. Salvatore et al. mentioned that IGs prepared with H_2_O_2_ exhibit a maximum broadening of the (002) peak compared with other oxidants [23]. IGs maintain the two main diffraction peaks: (002) and (004), around the original positions; this suggests that the graphite lattice is barely retained after the chemical processing. The intensity ratio of NGr and IGs (I(002)/I(004)) was calculated and plotted in the square in Figure 3a, demonstrating a notable decrease in this ratio after the intercalation process. This value can be considered an indication of positional disorder caused by the graphite layers’ expansion or exfoliation due to the insertion of intercalants [28]. Unmilled samples show a remarked reduction of crystallinity which is partially recovered with milling until this array is almost reached with the 30–1 sample. This behavior can be related to intercalated species’ concentration, as seen in the SEM-EDS section. On the other hand, the peak shifting is caused by the increasing interlayer spacing of the samples, suggesting that different levels of oxygen-containing groups were attached to the graphite lattice, as mentioned by Krishnamoorthy et al. [29]. A peak usually located around 10 to 11° is absent in our prepared IGs because this signal is generally correlated to an oxygen-carbon chemical interaction that occurs during strong graphite oxidation; colloquially, this compound is called graphite oxide or graphene oxide (GO) [8,22,30], the extinction of the (002) line implies complete graphite oxidation [31,32]. Additionally, the 10° peak is correlated to the creation of abundant oxygen functional groups located on the surface of the graphitic layers [33], which is not our case. M.J. Yoo mentioned that H_2_O_2_ additions normally reduce GO; thus, the high concentration of hydrogen peroxide used in this route could inhibit GO generation [16]. Additionally, a small peak can be seen at 42–43°, which can be attributed to a turbostratic presence of disordered carbon materials [19]. From the widening observed in the plane (002), the corresponding FWHM was calculated (Figure 3b), and we can notice that this value is increased as the milling time is increased; this behavior resulted from some factors: the intercalation of oxygen functional groups such as epoxy, hydroxyl, carbonyl, and carboxyl groups during the chemical reaction with carbon conjugated with the drawdown of the crystalline structure and defects generation during the HEBM [34]. Some authors described some possible causes of the chemical broadening of the IGs: Bannov et al. mentioned that after chemical intercalation with sulfuric acid, the XRD spectrum of IGs contains a mixture of two peaks: one corresponding to pure graphite and the other shifted to lower angles d (002) = 0.3368 nm with a lower interlayer distance, corresponding to an intercalation compound described as graphite bisulfate in the form of an asymmetrical peak appearing in the spectrum below 26° [12]. On the other hand, Hou et al. stated that some (002) diffraction peaks appearing at 26.59 and 26.73° suggest that some acid species remain in the interlayer spaces as residual compounds damaging the regular stacking of the graphite layers [2]. It is important to mention that ball milling was used to induce a uniform mixing of graphite and the intercalation agent favored by the mechanical action of the impact of milling media, but under higher milling times, graphite has a restacking effect resulting in increased intensity of the characteristic peaks [35], as can be seen in sample 30–1, where we can notice a reduction of FWHM and peak shifting to the almost original position. Meanwhile, sample 30–2 does not follow this behavior because the high peroxide concentration generates an increased chemical reaction, inhibiting the restacking effect.

**Figure 4:**
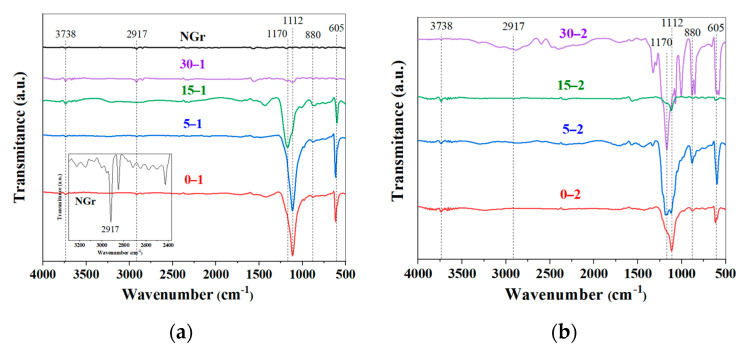
FTIR spectra of raw graphite and intercalated materials: (**a**) 30% and (**b**) 50% H_2_O_2_.

Fourier transform infrared spectroscopy (FTIR). Elemental analyses and DRX characterization show the presence of oxygen in the IGs, but the exact nature of the functional groups generated during processing is unclear. Through FTIR is possible to check and differentiate these chemical species based on their specific vibrational excitation modes by absorption of infrared light energy. In the IR spectra of Figure 4, NGr presents a relatively flat curve with no apparent observable peaks. Still, a magnification shows a strong peak at 2917 cm^−1^ correlated to the asymmetric stretching vibration of -CH_2_- groups as the base of its graphitic structure. On the contrary, the IGs samples exhibit strong bands associated with oxygen-containing functional groups after the chemical processing, evidencing that graphite was effectively functionalized by employing the couple H_2_SO_4_–H_2_O_2_ without any KMnO_4_ use [27,36]. The O-H vibration band is located in the region of 3600–3300 cm^−1^, which is attributed to the missing hydroxyl groups of residual water between graphite layers [30]. However, a strong band between 1300 and 1000 cm^−1^ indicates that large amounts of O-containing functional groups are linked to graphite [33]. The intense absorbance peaks at 605 and 1170 cm^−1^ correspond to the keto form, and the C-O vibration mode of the enol form is present, indicating the introduction of polar groups in graphite [11,32], suggesting an interaction of oxygen bonded to a carbon backbone (C–O) [37].

Meanwhile, stretching vibrations in the C=C plane of the unoxidized graphitic domain sp^2^ bonds are presented as weak signals at 1562 and 1628 cm^−1^ [25,37,38]. In IGs samples with low milling time (0 and 5 min.), there is a peak at 1112 and 880 cm^−1^ which correspond to vibrations modes of -OH bonds, that widens and moves to 1170 cm^−1^ denoting an induced (C-O) oxidation process promoted by milling. This is clear evidence that the molecular structure of powders largely depends on the preparation conditions [39].

**Figure 5:**
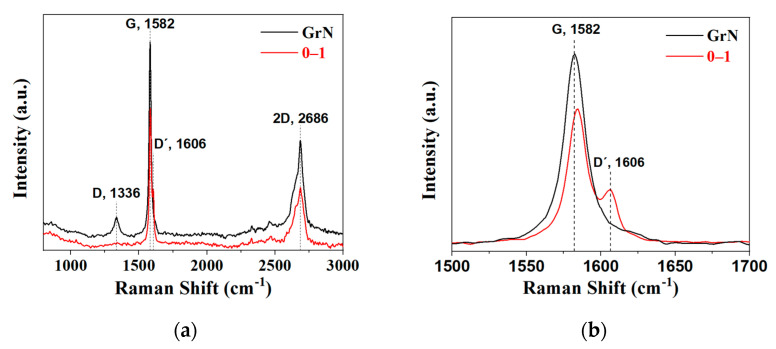
Raman spectra of raw graphite and the intercalated material (**a**) and magnification on the G band zone (**b**).

Raman spectroscopy. This technique is based on the inelastic scattering of molecules irradiated by monochromatic laser light. Raman measurements were carried out to determine the density of defects introduced during the intercalation process. The spectra of Figure 5a present the characteristic peak configuration of graphitic material, such as a strong G peak located at 1580 cm^−1^, which is related to in-plane C-C symmetric stretching vibrations and is associated with the sp^2^ carbon atoms, a small band at 1336 cm^−1^ is usually called the D band, which is related to sp^3^ carbon atoms vibrations and presence of defects in the graphitic structure in the form of vacancies, edge disorder, and defects. Another important feature is the presence of the 2D band located at 2686 cm^−1^ (referred to as 2D or G0 band), which is derived by the overtone of the D band as a result of an out-of-plane vibration mode, giving information about the structural orientation along the c-axis. The spectrum of NGr and the intercalated sample show differences in all bands. Typically, an intense oxidation process generates predominant structures by inserting fully oxygen groups on the basal plane and edges of graphite lattice (graphite oxides); their Raman spectrum shows two intense peaks of D and G bands with almost the same intensity [39,40,41]. Our intercalated material has not this behavior because graphite oxides are not generated following this route; this is in agreement with the evidence found in X-ray patterns (absent of the characteristic peak located at 10° in Figure 3). Yoo et al. [16] established that intensity reduction of the D band also occurs when the distance between defects is nanometric. The intense G band in the intercalated sample indicates a large percentage of the graphitic 2D hexagonal lattice structure formed by the union of -CH_2_- bonds (in good agreement with the evidence found in FTIR studies). Additionally, the G band has no appreciable widening effect, which is generally found in graphites with an extensive oxidation treatment [38]. It can be noticed a position shift of the G peak towards a higher wavenumber between samples (Figure 5b) can be related to distribution differences between inserted oxygenated functional groups (hetero-atoms) in the material [42]. Intercalated species also cause graphitic structural defects, inducing the appearance of a shoulder next to the G band; this signal is called the D’ band (located at 1610 cm^−1^); it was related to changes in the Fermi level [43]. The intensity of the 2D band is also lower for the intercalated sample, which can be attributed to defects in the graphitic layer arrangement due to the breaking of the stacking order in the c-axis derived from the chemical reaction and more graphene-like structure by the presence of the sp^2^ bonds [44]. Furthermore, there is a small shifting effect of the 2D band; it was related to the presence of oxygen-containing functional groups, which prevents the graphene layer from stacking correctly [19].

**Figure 6:**
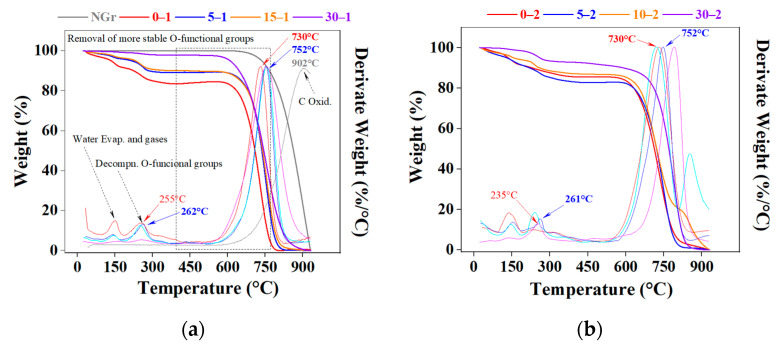
Thermogravimetric (weight loss) and derivate curves of raw graphite and intercalated materials: (**a**) 30% (series 1) and (**b**) 50% H_2_O_2_ (series 2).

Thermogravimetric analysis (TG). These studies followed the thermal behavior of NGr and IGs, looking for their stability and decomposition features (detected through the TG curves) [40]. In Figure 6a, the NGr sample has an almost flat curve without significant weight variation; at high temperatures (800 °C), it recorded a mass loss of only 10%. Thus, raw graphite presents a high resistance against oxidation, forming two main products, CO and CO_2_ (both gaseous), depending on the relative oxygen concentration. On the contrary, IGs show evident weight variations related to intercalated species presence (due to evaporation or decomposition) combined with carbon combustion, generating the intrinsic expansion phenomenon of IGs [33]. The first significant weight loss is observed at temperatures between 150 and 300 °C, commonly associated with water evaporation (steam) and gases released from the most labile functional groups [25]. Botas et al. [42] assumed that this phenomenon is generated in a sequential form: beginning with the releasing of a small amount of water at the initial heating stage and followed by a dramatic loss at 150–300 °C, these events correspond to the decomposition of oxygen functional groups slightly attached to the carbon structure, here the decomposition products are mainly H_2_O (g) and CO_2_ (g). Later, between 400–950 °C, there is a slower mass loss, and it is generally attributed to the removal of more stable oxygen functional groups, corresponding mainly to H_2_ and CO gases. Salvatore et al. [23] assumed another chemical mechanism of expansion related to the reaction between the principal intercalating agent and graphite reacting according to the following chemical equation:

C (*s*) + 2H_2_SO_4_ (*l*) → CO_2_ (*g*) + 2SO_2_ (*g*) + 2H_2_O (*g*)

where three moles of solid and liquid precursors release five molecules of products in gas form, generating this dramatic expansion observed in all tested IGs. Commonly, thermal stability is a property related to the intrinsic ability of a material to endure high temperatures without thermal degradation. As seen in the thermograms, milling time and peroxide concentration substantially affect the thermal stability of samples. The thermal decomposition located around 200 °C differs from sample to sample. Whereas NGr is stable at this temperature (showing no weight changes), samples prepared with H_2_O_2_ 30% has an average value of 258.0 ± 2.2 °C and the 50% samples present a lower value of 248.3 ± 7.8 °C, losing weight early (0–2 sample exhibits a peak at 235 °C), this means a difference of 26.6 °C compared to its 0–1 sample counterpart.

Consulted literature suggested that the ratio among different functional groups, especially epoxies and hydroxyls, influents the thermal stability; it is mentioned that OH groups produced are responsible for the enhanced thermal stability because hydroxyls are more difficult to decompose and are more stable than epoxy groups [32,45].

In addition, Mahmoud et al. reported a general decrease in thermal stability as the milling time is increased due to factors such as particle size, surface area, and compositional differences between samples induced by milling [18]. It is mentioned that the functional groups’ amount, type, and location can be altered by modifying the preparation conditions and strongly influencing their reactivity [42].

### 2.2. Graphite Pre-Expansion

**Figure 7:**
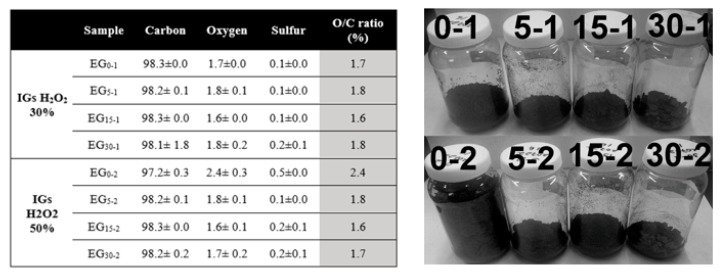
Elemental analyses performed on expanded graphites (in weight %), carbon/oxygen ratio, and a comparative image of prepared IGs. Series one corresponds to EG prepared from IGs using 30% hydrogen peroxide solutions (series two were obtained using H_2_O_2_ 50%).

As mentioned, hydrogen peroxide induces a room-temperature expansion, originating from the gas’s rapid pressure rise within the graphite interlayer array. This phenomenon is related to the H_2_O_2_ decomposition mechanism at low temperatures, producing H_2_O and O_2_ due to peroxide instability [23]. Two factors are needed to fulfill the particle pre-expansion: the presence of released gas between graphite layers and the lower relative surrounding environmental pressure [46]. As can be seen in the image attached to Figure 7, all IGs present the effect of room-temperature expansion to some extent; this effect is magnified with unmilled samples (0–1 and 0–2); in the image, each container has a complete batch of each run (~2.5 g of dry IG). The 0–2 sample exhibit the highest expansion due to its high H_2_O_2_ concentration during its synthesis, meaning a more significant availability of oxygen and bigger (unmilled) graphite particles available to expand. Additionally, under high-temperature exposition, IGs exhibit a massive and “spontaneous” volumetric expansion [47], preferably performed along one crystallographic axe; this process occurs due to the rapid vaporization of incorporated water [42] and intercalated species (O-containing groups), which were merged between the carbon sheets during the intercalation [8,10], these compounds forming folds from the separation of graphitic packed layers which provides channels to facilitate the transport of intercalation agents [11]. Some involved steps involve oxidizing and intercalation reactions for expansion forming graphene worms [48] or vermicular structures. This treatment is also called thermal reduction because it removes the O functional groups from the IGs [21,22,31]. The thermal reduction has advantages over chemical reduction because it is simpler and easier to perform, and there is no need to use additional chemicals or liquids [42]. The method efficiency is confirmed by the results of Figure 7, which shows the elemental composition of the IGs after the expansion process (30 s); as can be seen, there is a notable reduction in O and S contents of 70% and 93% on average, respectively. The series 1 (H_2_O_2_ 30%) samples kept an almost constant low O/C ratio. Meanwhile, series 2 follow a downward trend; thus, the heating process acts as a physical reduction process. On the contrary, as seen in the thermogram of Figure 6a, untreated graphite (NGr) has no expansion capacity under heating. Usually, the expansion process of IGs is conducted by putting the IGs in a furnace at a high temperature (~1000 °C) for a period below one min, leading to the decomposition of the IGs, generating a porous, cellular structure [12].

**Figure 8:**
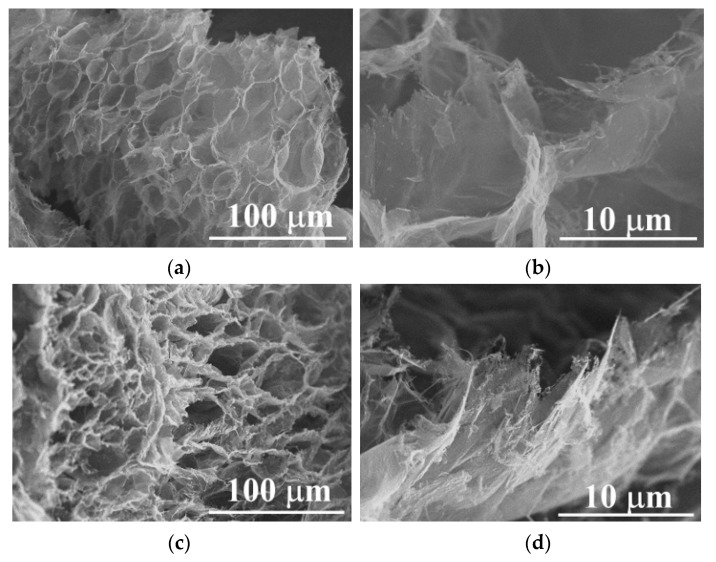
SEM micrographs of expanded samples (**a**,**b**) EG_0-2_ and (**c**,**d**) EG_5-2_.

Unfortunately, this expansion process is typically performed by high temperature or irradiation, leading to significant energy consumption [46]. The EG synthesis described in this work must be based on a greener alternative for graphite expansion, considering factors such as short reaction times, low investment costs, and high equipment availability. Based on the above, microwave heating was chosen because, under this type of irradiation, the IGs rapidly expand [48] due to their uniform heating features [21]. The microwave heating mechanism is related to the close conduction and valence bands for graphite which determines that the π-electrons move freely and can be accelerated by the electric field component of microwave radiation. Here, the heating model of Joule establishes that free electrons will collide with carbon atoms generating heat locally. Additionally, the functional groups and defects present on the surface and porous structures attenuated the incident microwaves, transforming them into additional heat; thus, the energy conversion from microwave to heat is further enhanced [49]. In Figure 8, it is noticeable that after the microwave expansion treatment, the expanded samples present remarkable changes in their morphology, reaching the peculiar three-dimensional structure accordion-like, with an important concentration of pores complemented with internal channels along the graphitic layers. These morphologies at low magnifications are similar to those obtained using the conventional heating process using the traditional route with a furnace oven [11,27,31,35,42,50].

### 2.3. Microwave Time Optimization

**Figure 9:**
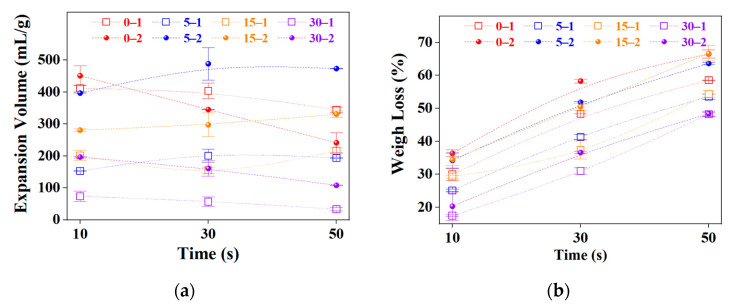
EV variation (**a**) and mass loss (**b**) during microwave heating as a function of time and peroxide concentration. Top series 2 (50%) and bottom series1 (30% H_2_O_2_).

IGs were processed using microwave radiation using three different time intervals from 10, 30, and 30 s. As a result of the heating process, the samples were expanded, and the so-called expansion volume (EV, mL/g) was determined for comparison. EV is an important parameter for adsorption efficiency because the material capacity increases as this parameter rises. There was mentioned that increasing EV values constitute a clear strategy to improve material performance further [1]. It is commented that oil adsorption capacity depends on not only the surface area but also the pore structure of the material since there is a strong dependence on EV and worm lengths [51]. Figure 9 shows the reached EV as a function of microwave heating time, and peroxide concentration, the samples prepared with 50% H_2_O_2_ concentration achieved almost 500 mL/g (5–2); meanwhile, with 30% of peroxide concentration, the sample with the highest value was 0–1 reaching 400 mL/g, as can be seen in the Figure 9a, the mentioned evidence suggests that samples prepared with more concentrated peroxide reach higher values compared to the lower concentration counterparts. As a clear comparison of the reached highly efficient process, other works reported values of 150 [48], 11.4 [8], ranges from 17 to 62 [12], and 110 mL/g [35]. As can be noticed, most of the samples presented an optimum EV after 30 s of microwave heating. Due to the fast temperature increase, mass loss can be directly correlated to the intercalant groups vaporization and carbon combustion; both reactions increase the pores and internal channels generation available for oil adsorption, increasing the oil removal capacity of samples, as can be seen in the next section. However, as microwave heating was performed under an air atmosphere, the incandescent material had contact with oxygen, and there was an additional weight loss due to carbon oxidation resulting in CO_2_ generation; all the samples presented a considerable mass loss with further microwave heating, as can be seen in Figure 9b. This effect is perceptible through the SEM images obtained at high magnifications (Figure 8b–d), where the borders of the graphitic layer are noticeable burnt.

EV and mass loss values are lower with IG products obtained with extended ball milling times (15 and 30 min). This can be related to their lower intercalant concentration (Figure 2, O-S concentration) and reduced particle size, which produce short worms compared to unmilled or slightly milled samples (0 and 5 min). Based on these plots, the appropriate ball milling time was set to 5 min. This negative effect of intensive milling was reported before with other IGs [35].

### 2.4. Oil Adsorption Capacity (OAC)

**Figure 10:**
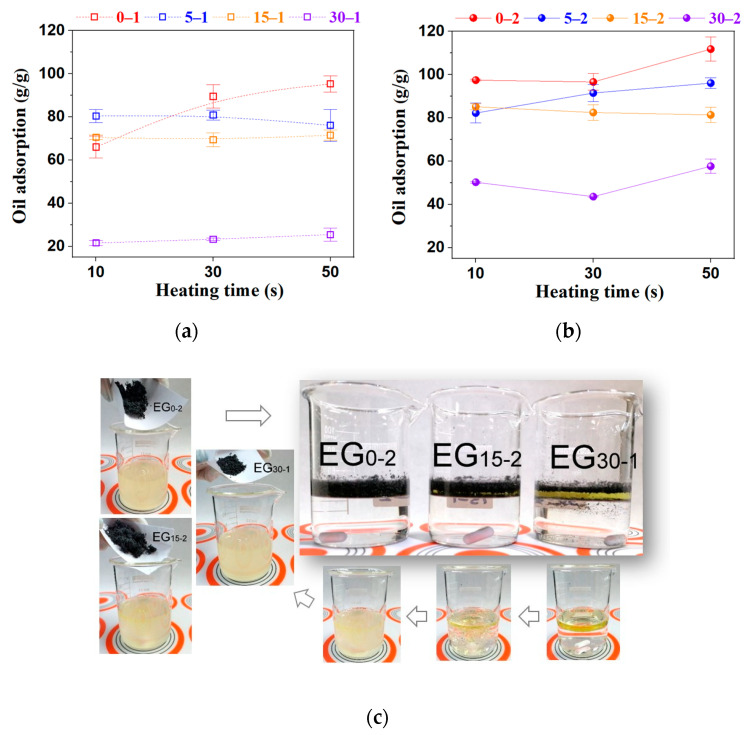
Graph of adsorption capacity as a function of the microwave heating time (**a**,**b**) and images of oil adsorption testing (**c**), EGs (50 mg) were added to a stirred diesel–water mixture to show EGs high oil selectivity; central photos are the samples after resting.

Diesel is an organic mixture of hydrocarbons roughly composed of 75% saturated alkanes (linear and monocyclic) plus ~25% aromatics (naphthalenes and alkylbenzenes) [52] which can be used as a reasonable model for adsorption experiments and lab testing, simple mixtures of diesel plus water can reproduce numerous oily and wastewaters. Prepared EGs were checked for OAC by directly adding the powders to a contaminated testing fluid prepared with diesel fuel and tap water. The addition of EG on the oily upper layer generates the immediate generation of an agglomerate formed by oil attached to the carbonaceous material. This behavior is caused by several absorbing spaces related to wrapping constructed by EG stacking on each other and pores located in each worm-like segment [11]. EG works as a selective oil adsorbent because of its peculiar network pore structure, weak polarity, and hydrophobic and lipophilic nature; additionally, EG does not mix with water even with agitation, showing a high selectivity to large organic compounds with weak polarities, such as oils. After the EG addition, the yellowish oily layer almost disappeared in a min and the contamination was no longer visible; this behavior is displayed in the images of Figure 10c. In a general way, evidence shows the high effectiveness of chemo-thermal processing, expecting to increase the overall performance of oil adsorption [6]. The plot of Figure 10a,b shows a clear tendency between the milling time, peroxide concentration, and microwave heating time on the OAC. Regarding milling, the highest OAC values were also obtained with the unmilled and slightly milled EGs (0 and 5 min). Samples milled for 15 min display an almost constant performance, and the further milled EGs (30 min) showed the lowest values. Thus, prolonged milling negatively affects the performance of the EGs for oil absorption.

**Figure 11:**
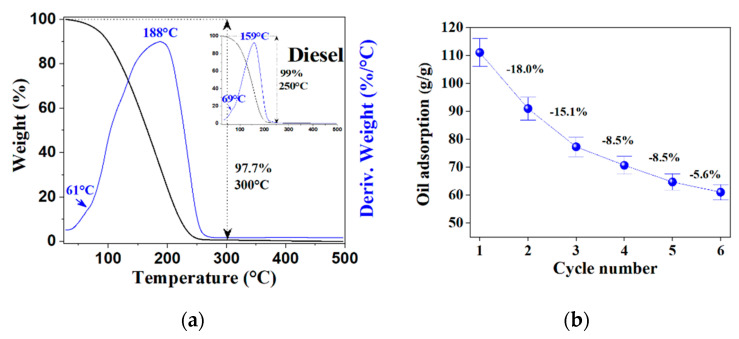
Thermograms with weight change and its derivate of saturated EG_0-2_ sample and pure diesel thermogram used as a comparison (**a**) and recyclability plot of EG_0-2_ over multiple cycles (**b**).

For peroxide concentration, higher H_2_O_2_ addition means better performance in all samples; this can be related to the superior porous formation and worm lengths because these features can provide more transport channels, expecting rapid adsorption rates [11]. It is mentioned that the micropores’ presence can increase the adsorption capacity of porous carbon materials [7], but the evidence contradicts the general assertion that adsorption efficiency depends substantially on the smaller particle size of the adsorbent [4]. Microwave heating time also affects the OAC, reaching an optimum with EGs treated for 30 and 50 s, depending on the sample; 10 s of microwave heating is not enough to achieve the full material expansion, and the overall performance of oil adsorption is negatively affected. The highest OACs were obtained with 0–2 and 0–1 EGs reaching values of 111.8 ± 5.6 and 95.3 ± 3.8 (g/g), respectively. A large sorption capacity, fast performance, and high selectivity are requirements for good adsorbates. Still, eco-friendly materials additionally must complain with reduced environmental impact and regeneration abilities to meet the recycling cycles [2] because the main drawback associated with adsorption technology is the adsorbent cost which elevates the overall treatment process cost. Current practices such as circular economy, sustainable development, and green chemistry-based engineering processes involve reusing and recycling industrial wastes and adsorbents [6]. Thus, one important operational parameter of adsorption processes is the adsorbent regeneration because recycling reduces further use of chemicals to preserve the environment [4,5]. In other words, not only must EG remove oil from water in an efficient way, but also both the oil and EG must be recovered and recycled correctly; unfortunately, EGs’ porous structure can be easily destroyed during the regeneration process [2]. To separate oil from the saturated EG, some process has been described but have many disadvantages: using filtration, the recovered amounts of oils decrease rather markedly with increasing recycling times due to the oil retention in the pores or on the surface of EG [53]. The manual squeezing of EG contained in pillows is a tedious and not-scalable process; it generates a loss of particles from the pillow and a decrease in the absorbent capacities after each cycle due to changes in the structural and morphology of the particles [1,27]. Oil recovery with compression was about 30% and decreased with increased regeneration cycles [2]. Another practical separation method is chemical extraction, but the solvents usually present higher toxicity than EG; thus, a mandatory full remotion is required to increase the investment, time, and process costs. To keep the porous microstructure of the EGs, a heating process for oil separation was tested in this work. The recyclability evaluation was performed with diesel fuel (boiling range 170–340 °C) and compared with an EG sample saturated after the oil adsorption test (EG0-2 OAC = 97 g/g) in a simultaneous TG-DTA equipment using air as an experimental atmosphere and a heating slope of 10 °C/min; the thermal curves are shown in Figure 11a. In the thermogram of pure diesel, there are two peaks in the derivate of weight change against temperature (blue curve), one located at 69 °C which is related to the volatilization of lightweight components, and the second one located at 159 °C associated with diesel evaporation; those generate the characteristic double peak as can be seen in the attached thermogram of pure diesel used as reference. At 300 °C, diesel is separated from the oily saturated EG, leaving the absorbent as residue. The weight change (97.7%) corresponds to the adsorbed oil near the original saturation level of the EG0-2 sample.

Hence, diesel can be removed using distillation as a separation process without disturbing the porous features of EGs. Then, 50 mg of fresh EG was saturated with the diesel–water mixture; the oiled EG was separated, weighted, and left at 250 °C in a thermobalance until there was a variation < 2 mg/90 s. The residue was cooled down until room temperature and added to a new batch of diesel/water mixture to absorb the oil; the process was repeated five times to check the EG recycling capability; the results are shown in Figure 11b, where the AOC was calculated taking in account the weight of the recovered EG (in dry basis). In the first cycle, the adsorption capacity was 111 g/g. As might be expected, AOC decreases with the recycling process, as can be noticed in the plot. After six cycles, a stabilization trend can be observed. The oil yield recovery was >95% during the recycling process, indicating that oil is physically adsorbed in the EG due to its reversible characteristic [54].

**Figure 12:**
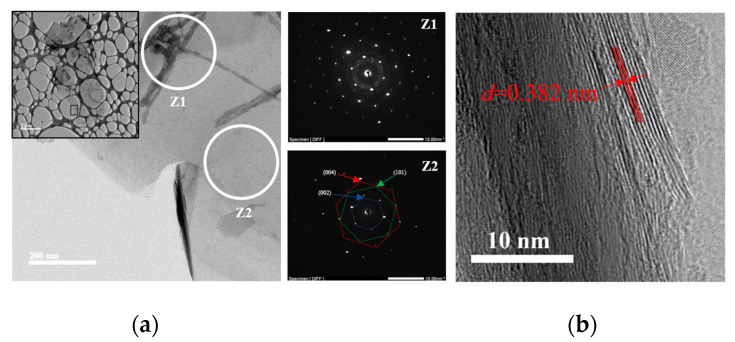
TEM micrographs and SAED patterns of the 0–2 sample after the expansion process (**a**) and HRTEM image with an interplanar measurement (**b**).

Transmission Electron Microscopy (TEM). Using TEM, it is possible to study morphology and crystalline degree through selected area electron diffraction (SAED) patterns of the expanded graphite sample. Figure 12a shows a graphite particle with different levels of transparencies attributed to the different number of layers [29]. It is noticeable high transparency because it is made up of a few numbers of graphitic layers. Additionally, two different primary morphologies are noticed; to clarify this, two SAED patterns were obtained from the same figure. The Z1 pattern, taken from a semi-transparent zone, presents clear diffraction spots from different crystalline planes that are consistent with the hexagonal lattice of graphite [25]. Meanwhile, Z2 offers a combination of high crystallinity attributed to the increased transparency zone as Z1 and polycrystalline diffraction rings with multiple spots formed by small graphite sheets with different orientations [55]. Moreover, it can be noticed that the ordered graphite in the high-resolution TEM micrograph (Figure 12b) with a clear lattice pattern presents an expanded interlayer spacing of 0.382 nm higher than the average graphite parameter (0.335) because staking defects caused by the thermochemical process, confirming the graphite expansion (being consistent with the results presented in DRX section) [56].

## 3. Discussion

EG is not a recent product; Brodie prepared it in 1859 by adding potassium chlorate to a graphite slurry in fuming nitric acid. In 1898, Staudenmaier improved the reaction using a mixture of concentrated sulfuric and fuming nitric acid with chlorate. C. Schafhäutl and B. Brodie (1851) described the graphite intercalation based on the following reaction scheme:

$$24nC+O_{x}^{z}+mH_{2}SO_{4}\dot{\to }C_{24}^{+}\cdot HSO_{4}^{-}\times \left(m-1\right)\;H_{2}SO_{4}+HO_{x}^{\left(z-1\right)}$$

where *O_x_* is the oxidizing agent and *C* is the carbon atoms in the graphite. The stage and kinetics of bisulfate formation depend on the sulfuric acid concentration and the type of oxidizing agent involved [23]. Hummers (1958) reported the most common method today: graphite oxidation with *KMnO*_4_ and *NaNO*_3_ in concentrated *H*_2_*SO*_4_ [16,21,26,32,36,38,57]. The first methods involve very long reaction times (days) and have certain risks of self-ignition or explosion.

For these reasons, the Hummers’ method is the most widely used nowadays due to its safety and simplicity [20]. It is reported that H_2_SO_4_/HNO_3_ mixture acts as a scissor and drill in graphene planes facilitating the oxidation solution penetration; KMnO_4_ induces complete intercalation of graphite (forming graphite bisulfate); thus, the addition of HNO_3_-NaNO_3_ is unnecessary [30]. James M. Tour introduced a “safer alternative”, omitting the usage of nitric acid through an oxidation mixture of potassium permanganate, concentrated sulfuric acid, and phosphoric acid [22,38,47]. Noted that the three first procedures involve the generation of toxic gases such as NO_2_, N_2_O_4_, or ClO_2_ (highly explosive) [25,30], also an indiscriminate use of H_2_SO_4_ as an intercalant agent, which can cause serious pollution issues [46] and all of them require massive use of high purity washing waters to purify the resulting product. It is important to point out that heavy metal pollution due to the indiscriminate disposal of wastewater is a global environmental concern because wastewaters frequently contain toxic heavy metal ions [33]. Metals with a high atomic number and a density greater than 5.0 are toxic and are termed heavy metals; this definition includes some transition metals, such as manganese, which has been catalogued as neurotoxic [58]. Thus, it is mandatory to eliminate heavy metal ions in wastewater before it is released into the environment, these can be removed by precipitation, membrane filtration, sorption, and ion exchange, but the cheapest and simple method for its elimination is the use of low toxicity alternative chemicals following the green chemistry principles.

**Figure 13:**
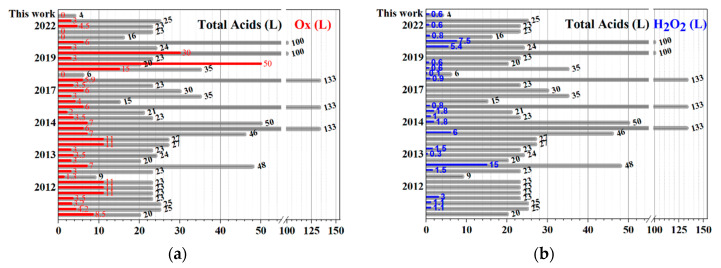
Comparative graph with values taken from Table 1, total oxidants (**a**) and hydrogen peroxide (**b**) used during the product synthesis.

Through this reasoning, to avoid the use of metallic oxidants (permanganate as a source of manganese) and to reduce the quantity of washing water, the alternative use of hydrogen peroxide (H_2_O_2_) is proposed. H_2_O_2_ is used as a strong oxidant due to the highly reactive properties of its unstable peroxide bond; the main by-products of its decomposition are water and oxygen (both low-impact and greener compounds). Additionally, hydrogen peroxide is a good alternative for graphite reactions because peroxide causes a large number of π-conjugated carbon radicals by reacting hydroxyl anions to the double bonds of the disrupted π-conjugated plane of the graphitic molecule [16]. The effect of H_2_O_2_ in the Hummers method has been neglected. It is arbitrarily used only to remove MnO_4_^−1^ because its exact proportion was not specified [16]. On the contrary, S. Ho reported evident variations in EV with the molar ratio H_2_SO_4_/H_2_O_2_, which affects the graphite intercalation [17]. The revised references summarized the type of chemicals and experimental procedure to build a comparative chart (Table 1). The following experimental parameters were taken into account: processed graphite per run (in Kg), type of used acids (H_2_SO_4_, HNO_3_, H_3_PO_4_, etc., in L), type of oxidant agent (in the form of metallic salts such as NaNO_3_, KMnO_4_, KCLO_3_, K_2_Cr_2_O_7_, NaIO_4_, etc., in Kg), used hydrogen peroxide (expressed as H_2_O_2_ 100%, in L) and the process water used for purification was not taken into consideration due to space constraints, but in some references, this value reaches 600 mL per gram of graphite processed, (present route uses 80–100 mL). Finally, the sum of the chemicals (concentrated acids, oxidant metallic salts, and hydrogen peroxide) was calculated based on a Kg of initially processed graphite. Figure 13 and Table 1 show that twenty years ago, the proportions of chemicals used for the graphite intercalation were kept almost unaltered following the classic Hummers method early described in 1958. In contrast, the defined process eliminates the use of oxidants salts such as permanganate, nitrate, or dichromate (cero vs. seven parts on average per part of graphite, see the average value in Table 1), the proportion of concentrated acids (four vs. almost thirty-eight parts in average) is the lowest of all consulted references, even the consumption of hydrogen peroxide is strongly reduced, with this critical reduction in the used chemicals and their amounts the consumption of washing waters are kept to a minimum, obtaining a pure product without any metallic salts remnants. Finally, the performance as an absorption agent for oil removal stands out among the described in the consulted literature.

## 4. Experimental Procedure

EG was produced using a modification of the classical Hummers route, using a reduced chemical consumption of intercalation agents and fewer chemical steps.

### 4.1. Materials

The used precursors were natural graphite flakes (Alfa Aesar, 99.9% purity, −10 mesh), concentrated sulfuric acid (JT Baker, 98% purity), and hydrogen peroxide solutions (Sigma Aldrich 30 and 50% w/w in H_2_O), both in reactive grade. Commercial diesel fuel was used as a model oil for adsorption testing; it was purchased from the official national fuels distributor (PEMEX).

### 4.2. Intercalated Graphite (IG) Preparation

This material was prepared by processing a mixture of 10 mL of concentrated sulfuric acid with 2.5 g of graphite flakes (NGr) in a SPEX 8000M mill during four-time intervals (0, 5, 15, and 30 min) using a hardened steel vial and six balls (3 × 13 mm and 3 × 10 mm in diameter). The milled paste was carefully put in a 150 mL baker and was introduced in a cooling bath at –5 °C for 5 min to reach a temperature below 0 °C (the paste becomes solid). Then 3 mL H_2_O_2_ aqueous solution (50 and 50%) was slowly added from a burette avoiding any temperature increase. The reaction beaker was kept in the ice bath for 10–12 h. The intercalated product was washed with 200–250 mL of deionized water in a Buchner funnel, and the solid was dried in an oven at 60 °C for 12 h. Acid-washing waters were neutralized (pH 6–8) and discharged to the drain. Figure 2 shows the nomenclature used in this work for the original graphite and the intercalated samples.

### 4.3. Graphite Expansion

The dry IG was expanded using an alternative heating route based on a domestic microwave oven (Frigidaire, model FMDL17S4GLW) operated at 700 W using heating cycles of 10, 30, and 50 s (10 s heating/10 s resting). This method was chosen because, under microwave radiation, IG particles are rapidly expanded, and the solid reaches hundreds of grades in s, giving uniform heating [21]; this causes rapid evaporation of intercalated species, resulting in fuming and lightening obtaining highly porous worm-like structures [10,43,48,49].

### 4.4. Expansion Rate

This parameter was calculated following the equation:

EV(mLg)=Vm

where *EV* is the expanded volume, *V* is the volume (in *mL*), and m is the sample quantity (in g). In total, 100 mg of the IG samples were processed in the microwave oven for time intervals from 10, 30, and 50 s in a 50 mL Pyrex glass beaker; the reached expanded volume was marked in the beaker, and the EGs were weighted to check the mass difference. Deionized water was added to the empty beaker until the mark, then water mass was converted to its equivalent volume (using the water density at registered temperature). *EV* was calculated from three experimental runs with their respective statistical analysis (average and standard error).

### 4.5. Diesel Sorption Studies

Briefly, 20 mL of commercial diesel fuel was added to 40 mL of tap water in a 150 mL beaker at room temperature, the mixture was stirred for 20–30 s, and then, 50 mg of EG was added. After 30 min of resting time, the oil-saturated solid was separated using a previously weighted mesh (#100), and the difference was recorded. Diesel sorption capacity was determined by weighing the wet *EG* and expressed as the trapped oil weight per gram of adsorbate (g/g) in triplicate. The adsorbed oil (*AO*) per weight unit was obtained using the following formula:

$$AO\left(\frac{g_{Diesel}}{g_{EG}},\;\%\right)=\frac{\;M-M_{0}}{M_{0}}\times 100\%$$

where *M*_0_ and *M* are the weights of the *EG* before and after the absorption process, respectively.

### 4.6. Materials Characterization

Structural and phase identification studies were performed through X-ray diffraction (XRD) using a Panalytical X’Pert PRO diffractometer with Cu Kα radiation (λ = 1.5405 Å) operated at 40 kV and 30 mA; the diffractograms were obtained using a scanning 2θ ranged from 5 to 60° with a 0.008°/step and 60 s/step. The morphological and compositional studies of raw, intercalated, and expanded graphite powders were carried out via scanning electron microscopy (SEM) using a Hitachi SU3500 and a JSM-7401 F field emission microscopes. Wet route chemical analyses were performed with a ThermoScientific Flash Smart Elemental Analyzer and a ThermoScientific plasma emission spectrometer model iCAP 6500 Series (ICO). The identification of functional group and structure elucidation was made in a Shimadzu IR spectrophotometer model Affinity 1S. Raman spectrometry was performed using a LabRam HR VIS-633 microscope (HORIBA, Ltd. Miyanohigashi, Japan) equipped with a He-Ne laser source. Thermogravimetric studies were carried out in a simultaneous SDT-TG calorimeter from TA instruments (model Q600) and a TG calorimeter (model Q500); runs were performed from ambient temperature to 950 °C under a heating rate of 10 °C min^−1,^ and air atmosphere. Transmission electron microscopy (TEM) studies were performed using a Hitachi 7700 microscope (HITACHI, Tokyo, Japan), and high-resolution transmission electron microscopy (HRTEM) was carried out using a TEM JEOL JEM 2200FS (JEOL, Tokyo, Japan) microscope. For TEM preparation, a few mgs of the sample were placed in a glass vial with 5 mL of methyl alcohol and sonicated in a Branson Digital device (Danbury, CT, USA) for 5 min. A drop of the sonicated solution was taken with a capillary tube and deposited on a copper grid with a formvar-carbon membrane.

## 5. Conclusions

This green chemistry-based method reduces the consumption of concentrated sulfuric acid during the intercalation reaction to only 10% (compared to the average values consulted in the literature). Thus, the quantity of neutralization chemicals to treat effluents is notably reduced. Additionally, metallic oxidants in the form of alkaline salts of anions such as permanganate, chromate, or chlorate are entirely avoided. Several environmental advantages of the above are: the prepared EGs do not have toxic metal remnants in the form of Mn^+2^ or Cr^+x^ ions, there is also a notable reduction of water consumption used for the material purification, and the chemical process is simplified because only two chemicals were used following this ecological route: sulfuric acid and hydrogen peroxide. The expansion process was easy and low-cost, using microwave heating in a domestic oven to reduce energy costs. SEM-EDS analyses indicated a notable room temperature expansion, and intercalated products are composed only of carbon and intercalation elements (sulfur and oxygen). DRX studies show internal changes in the crystallographic network of graphite due to the presence of intercalated species, which were verified by FTIR (presence of oxygen functional groups) and Raman studies (changes and shifting of the characteristic graphitic D, G, and 2D bands). Thermograms show that the milling time and concentration of peroxide induce changes in the stability and decomposition features of IGs; after thermic treatment, the concentration of S and O was notably reduced. Thermal events such as vaporization, decomposition, and carbon combustion were observed during the calorimetric studies and microwave expansion, increasing the specific volume of samples. The highest EV value was reached with a 5–2 sample (500 mL/g), using higher peroxide concentration met the superior values with optimum times of 30–50 s depending on the sample. Prolonged milling time had a negative effect on the expansion and oil adsorption capacity of EG because of its lower intercalant insertion and small resultant particle sizes. EGs were tested for oily water remediation of artificial polluted water with diesel, showing high oil–water selectivity and rapid adsorption. The highest OAC was obtained with 0–2 and 0–1 samples, reaching values of 111.8 ± 5.6 and 95.3 ± 3.8 (g/g), respectively. Adsorbed oil was removed by heating, and EG recycling capability was checked using up to six cycles, where the initial efficiency decreased with recycling. TEM studies show that an EG sample was constituted by a few layers of graphene with high crystallinity and the presence of polycrystalline elongated particles. Additionally, the intercalation and expansion process increased the interlayer spacing to 0.382 nm.

## Figures and Tables

**Table 1:** Summary chart with the type of chemical and amount used to process one Kg of raw graphite, based on the description of each reference, noticed that H_2_O_2_ was only used to eliminate permanganate residues, not as a formal oxidant.

Year, [Reference]	Total Acids = (L)	Total Oxidants (Kg)	H_2_O_2_ (100%, L)
2009, [37]	20.0	8.5	
2011, [59]	25.0	4.2	1.05
2011, [8]	25.0	3.2	1.05
2012, [40]	23.0	3.5	3
2012, [31]	22.9 8.5	11.0 1.3	
2013, [30]	23.3	3.0	1.5
2013, [42]	48.0	7.0	15
2013, [47]	20.0	3.0	
2013, [60]	24.0	3.5	0.3
2013, [29]	22.5	3.0	1.5
2013, [61]	26.5 46.0 133.3	11.0 7.0 6.0	6
2014, [41]	50.0	7.0	1.8
2015, [39]	23.0	3.5	1
2015, [22]	21.3	2.0	1.8
2017, [19]	133.3	6.0	0.81
2017, [20]	15.0 35.0 30.0 22.5	4.0 3.0 6.0 3.5	
2017, [36]	133.3	5.9	0.9
2017, [7]	6.0	0.0	0.06
2017, [23]	35.0 20	15.0 50	0.6 0.6
2019, [16]	23.0	3.0	
2019, [28]	100.0	30.0	
2019, [62]	24.5	3.0	5.4
2019, [26]	100.0	6.0	7.5
2021, [17]	16.0	0.0	0.84
2021, [21]	22.9	0.0	
2022, [5]	23.0	4.5	0.6
2022, [2]	25.0	3.0	
**Average**	**37.8 ± 5.6**	**7.1 ± 1.5**	**2.6 ± 0.8**
**This work**	4.0	0.0	0.6

## Data Availability

Not applicable.
